# Comparison of the efficacy of acupuncture with tuina with acupuncture-only in the treatment of peripheral facial paralysis: a network meta-analysis

**DOI:** 10.1007/s11739-024-03562-2

**Published:** 2024-03-14

**Authors:** Xinyuan Deng, Hao Zhu, Luyan Shi, Yanting Li, Haiping Shi, Yicheng Wu, Yu Zhang

**Affiliations:** 1 The First Clinical Medical College, Anhui University of Chinese Medicine, Hefei, Anhui China; 2 The First Affiliated Hospital of Anhui University of Chinese Medicine, Hefei, Anhui China

**Keywords:** Acupuncture in combination with tuina, Acupuncture, Conventional meta-analysis, Network meta-analysis, Peripheral facial paralysis

## Abstract

At present, traditional Chinese medicine treatment is considered safe for treating peripheral facial paralysis (PFP). Acupuncture-only and acupuncture combined with tuina are widely used for this purpose. However, it is not clear whether acupuncture combined with tuina is better for treating PFP than acupuncture-only. Conventional meta-analysis and network meta-analysis were used to compare the clinical efficacies of acupuncture combined with tuina and acupuncture-only in the treatment of PFP. Randomized controlled trials (RCTs), with the subjects being patients with PFP and treatment interventions including acupuncture combined with tuina, acupuncture-only, tuina-only, placebo, single Western medicine, and steroids combined with other Western medicine were searched from both Chinese and English databases. The primary outcomes included Modified House–Brackmann (MHBN) scores and Sunnybrook Facial Grading System, whereas the secondary outcomes included cure time, Portmann scores, and physical function scale of Facial Disability Index, using conventional meta-analysis and network meta-analysis. The study included 22 RCTs with a sample size of 1814 patients. The results of conventional meta-analysis (MD = 16.12, 95%CI 13.13,19.10) and network meta-analysis (MD = 14.53, 95%CI 7.57,21.49) indicate that acupuncture combined with tuina was better than acupuncture-only in improving MHBN and shortening the cure time (MD = − 6.09, 95%CI  − 7.70, − 4.49). Acupuncture combined with tuina was the optimal therapy for improving MHBN (SUCRA was 100%) and shortening the cure time (SUCRA was 100%). The results of this meta-analysis indicate that acupuncture combined with tuina can significantly improve MHBN and shorten the cure time, compared with acupuncture-only. However, the current evidence is insufficient, and more high-quality clinical studies are needed.

*Registration:* This study had been registered with PROSPERO (CRD42022379395).

## Introduction

Peripheral facial paralysis (PFP), also known as Bell’s palsy or facial neuritis, is a nonspecific inflammation of the facial nerve in the foramina Stylomastoideum, which can result from a variety of causes. Clinically, most cases are acute, with the main manifestations being the disappearance of the nasolabial groove, the tilting of the corner of the mouth to the left/right, the disappearance of forehead wrinkles, and an inability to close the eyelid [1]. PFP affects approximately 25 people per 100,000 [2], and about 80% of patients require no medical intervention [3]. Modern medicine lists microcirculation disturbance theory, viral infection theory, immune theory, and neurogenic theory as the primary causes responsible for the pathogenesis of this disease [4]. According to traditional Chinese medicine (TCM), this disease was first recorded in the Yellow Emperor’s Inner Classic. It has been pointed out that PFP was mostly caused by the lack of healthy qi in the human body, and this deficiency adversely affected the head and face, causing the blockage of qi and blood, the obstruction of meridians and collaterals, and the onset of muscular dystrophy [5, 6].

At present, Western medicine mainly uses steroids and antiviral drugs to reduce the pressure on the facial neural tube and relieve facial nerve edema for treating PFP [7]. The drugs can be taken alone or in combination. For example, treatment with oral prednisone in the acute phase of PFP can help cure the local inflammation, and patients with a clear sign of viral infection can be simultaneously treated with oral acyclovir. However, oral steroids are associated with a risk of exacerbating digestive disorders, and people with underlying medical conditions can experience adverse reactions such as gastrointestinal ulcers [8]. TCM is widely used for treating PFP and is believed to be safe and effective [9–12] and acupuncture and tuina are most commonly used for this purpose. In the TCM theory system, qi and blood are the two fundamental substances in the human body, which play an important role in the life activities of the human body. At the same time, the meridians are significant channels for the body’s running qi and blood. Acupuncture can regulate qi and blood, promote inflammatory absorption, eliminate edema, and regulate nervous and immune functions [13–16]. Tuina therapy is associated with dredging meridians, improving facial nerve injury, and promoting nerve repair. Compared to oral Western medicine, acupuncture and tuina are not associated with comorbidities or contraindications and limitations of underlying disease because they are administered at the acupoints and on the surface of the body and are suitable for most patients. Patients might experience adverse reactions such as dizziness, palpitations, and tightness in the chest during manipulation [17] due to hunger and tension, but these adverse reactions can be avoided. Previous studies have demonstrated [18–21] that the application of acupuncture and tuina therapy for PFP can regulate the function of the cerebral motor cortex, accelerate the repair of the injured facial nerve, and promote the recovery of facial nerve function Zhao et al. [22] studied acupuncture combined with tuina in the treatment of PFP and found it to be significantly superior to conventional acupuncture in improving the conduction amplitude of the facial nerve. Although several clinical trials have investigated the efficacy of acupuncture-only and tuina-only in the treatment of PFP, the efficacy of acupuncture in combination with tuina compared with acupuncture-alone has not yet been investigated.​

In this study, we have comprehensively analyzed and compared the clinical efficacies of acupuncture combined with tuina and acupuncture-only in the treatment of PFP, using conventional meta-analyses and network meta-analyses.

## Methods

Only randomized controlled trials (RCTs) were included in the conventional meta-analysis and network meta-analysis performed in this study. Primary outcomes included Modified House–Brackmann (MHBN) scores and Sunnybrook Facial Grading System (SFGS). Secondary outcomes included cure time, Portmann scores, and physical function scale of Facial Disability Index (FDIp). This research had previously been registered with PROSPERO, with the registration number CRD42022379395.

### Search strategy

The RCTs dealing with either acupuncture, tuina, or acupuncture combined with tuina for treating PFP were obtained from Chinese and English databases, which included China National Knowledge Infrastructure (CNKI), Wanfang data, Chinese Science and Technology Journal Database (VIP), SinoMed, Medline, Embase, and Cochrane library. The databases were systematically searched from the time of inception until November 30, 2022. Keywords searched included “peripheral facial paralysis” (“facial paralysis” or “idiopathic facial paralysis” or “facial neuritis” or “Bell’s palsy” or “Bell’s facial paralysis”), intervention (“acupuncture” or “tuina” or “massage” or “manipulation”), and RCT (“RCTs” or “trial” or “trials”).

### Eligibility criteria

#### Inclusion criteria

1. Types of studies: Full text of the RCTs with data has been publicly published in China or other countries, in Chinese or English language.
2. Types of participants: Participants diagnosed with PFP meet the diagnostic criteria of “neurology,” “acupuncture therapeutics,” and “peripheral facial palsy” [23]. The patient’s age, sex, region, ethnicity, and duration of illness were not restricted.
3. Types of interventions: Acupuncture combined with tuina, acupuncture-only, and tuina-only were the main intervention measures for the treatment group, while acupuncture combined with tuina, acupuncture-only, and tuina-only along with placebo, Western medicine (taken alone or in combination), were the intervention measures for the control group.
4. Types of outcomes [24]:

Primary outcomes:

① Modified House–Brackmann (MHBN): MHBN is a graded scale aimed at assessing facial nerve function impairment, ranging from 0 to 100. A comprehensive score is used to assess facial nerve functions that include raising of the forehead, frowning, shutting of the eyes, nose shrugging, facial muscle strength, nasolabial folds, drumming of the cheeks, whistling, and tooth movement. The higher the score, the better the facial nerve function. A score of 100 indicates normal facial nerve function, 75–99 indicates mild facial nerve dysfunction, 50–74 indicates moderate facial nerve dysfunction, 25–49 indicates moderate to severe facial nerve dysfunction, 1–24 indicates severe facial nerve dysfunction, and 0 indicates complete loss of facial nerve function and complete paralysis of facial muscles. MHBN scale scores were evaluated before and after treatment, with higher scores indicating better clinical efficacy.

② Sunnybrook Facial Grading System (SFGS): SFGS is a comprehensive scoring method aimed at evaluating facial nerve function that grades the function of five peripheral branches of the facial nerve from static and dynamic links on a scale of 0–100. The eyelids, nasolabial fold, and mouth angle of the affected side of the patient at rest were used as observation points for scoring. With regard to dynamic time scoring, patients were scored separately based on the raising of their eyebrows, shutting their eyes, shrugging their noses, smiling, sucking their lips, and other movements. Higher scores indicated better facial nerve functions. A score of 100 indicated complete symmetry of facial movements and normal facial nerve function, whereas a score of 0 indicated inability to perform voluntary facial movements and complete loss of facial nerve function.

Secondary outcomes.

① Cure time: Cure time refers to the time from the first treatment to the complete recovery of facial nerve function. Shorter cure times indicated better clinical effects.

② Portmann scores: The Portmann scores is a scale that rates facial nerve functions, focusing on facial movements. Six facial movements (frown, shutting of the eyes, drumming of the cheeks, nose wrinkles, whistling, and smiling) were rated and scored between 0 and 20 points. A score of 20 indicated identical movement on the unaffected side, and a score of 0 indicated complete absence of voluntary movement on the affected side and complete paralysis of the facial muscles.

③ Physical function scale of Facial Disability Index (FDIp): The facial Disability Index (FDI) is divided into physical functioning score (FDIp) scale and social life functioning (FDIs) scale, which is a simple self-rating questionnaire for rating physical disability and psychosocial factors associated with facial neuromuscular function. The five aspects of FDIp include difficulty in eating, difficulty in drinking, difficulty with special pronunciation, excessive tears in one eye, and difficulty in brushing teeth. Patients are scored from 0 to 25 (score can be converted to the hundred-mark scale by using the formula: (raw score  − 5) *5), with a score of 25 indicating complete normal physical function related to facial neuromuscular function and a score of 0 indicating severe physical dysfunction.

#### Exclusion criteria

The following articles were excluded from the study:

1. Nonclinical multiarm RCTs include case reports, protocols, theoretical discussions, literature studies, patient before and after comparison, systematic evaluation, and expert experience literature.
2. Intervention measures were not exclusively acupuncture or tuina therapy or were combined with different treatment modalities such as moxibustion, Chinese herbs, and acupoint injections.
3. Outcomes of the study were not related to this study, effective data could not be extracted, or the outcome data were incomplete.
4. Republished articles.

### Studies selection and data extraction

The search results of different databases were imported into EndnoteX9 (Clarivate, Philadelphia, PA, USA) software. Two researchers (DXY and ZH) independently screened the literature based on the inclusion and exclusion criteria. The repeated studies were first excluded. Titles and abstracts of studies were reviewed, and studies that did not meet the inclusion criteria were excluded. The full texts of the remaining articles were reviewed, and the appropriate articles were finally selected. The two researchers reviewed the results of the selected articles with each other and, in the event of the lack of consensus, consulted with the third researcher (ZY) to discuss and solve the problem. After screening of the studies, study information and data were extracted, including first author, time of publication, treatment course, number of cases, ages of patients, intervention measures, outcomes, and adverse events. The data were recorded and summarized using Excel. In the event of a lack of consensus regarding the information and extraction process of the data, a third researcher was consulted to reach a consensual decision.

### Assessment risk of bias

Two researchers (SLY and LYT) independently assessed the assessment risk of bias using the “Risk of Bias” evaluation tool in the Cochrane Manual [25]. The specific items considered for risk bias assessment were as follows: ① random sequence generation; ② allocation concealment; ③ double blindness of subjects and researchers; ④ blindness of results; ⑤ loss of follow-up and withdrawal; ⑥ selective reporting; and ⑦ other bias. The included RCTs were evaluated for each item in terms of low risk of bias, high risk of bias, and unclear risk of bias. Two researchers independently evaluated and examined the results and in the event of a lack of consensus, further discussions were held, or a third party was approached.

### Statistical analysis

For statistical description, dichotomous variables were described in terms of odds ratio (OR), continuous variables were expressed in terms of mean difference (MD), and 95% confidence interval (CI) was used for interval estimation.

Review manager 5.3 (The Nordic Cochrane Centre, Copenhagen, Denmark) was used to support the risk of bias assessment of the included literature and for making a bias risk assessment figure. Review manager 5.3 was used for the conventional meta-analysis, and forest plots were obtained. Chi-squared test and *I*^2^ index analysis were used to verify statistical heterogeneity. If *P* > 0.1 or *I*^2^ ≤ 50%, a fixed-effects model was adopted considering the limited heterogeneity of the study. If *P* ≤ 0.1 or *I*^2^ > 50%, it indicated a higher heterogeneity of the included studies, and a random-effects model was used after analyzing the cause of the enhanced heterogeneity. Subgroup analyses were performed when exploration of clinical heterogeneity was necessary. Subgroup analysis was based on multiple factors: The first factor included demographic features such as race, gender, age, and regional differences. The second factor was the difference in the approach to implement intervention measures, such as whether to treat PFP in acute palsy and whether to treat PFP based on its stage. The third factor was the variation in evaluating outcomes, such as whether an outcome was using a hundred-mark system or a non-hundred-mark system, which would require numerical conversion if necessary. The stability of the conventional meta-analysis results was explored by performing a sensitivity analysis.

The network meta-analysis was performed using STATA 14.0 (Stata Corp, College Station, TX, USA), and the network plots and surface under the cumulative ranking (SUCRA) were constructed. Direct and indirect comparisons were made between the interventions. ADDIS v1.16.8 (Groningen, the Netherlands) was used for evaluating the potential scale reduction factor (PSRF). The convergence of the iteration was considered to be good for PSRF values between 1.00 and 1.05, with the convergence of the iteration considered to be better with values closer to 1, which could be obtained by using the consistency model analysis to obtain more reliable results. The network meta-analysis was accomplished using the consistency model, and the inconsistency test using the point method was performed in the event of the existence of a closed loop between the interventions. The consistency model was used for the analysis for *P* > 0.05, otherwise, the inconsistent model was used. The SUCRA of each intervention was ranked. A larger SUCRA of MHBN, SFGS, Portmann scores, and FDIp corresponded to a higher probability of the corresponding intervention being the best treatment. A smaller SUCRA of the cure time corresponded to a greater probability of the corresponding intervention being the best treatment. The results of the network meta-analyses have been presented in the tables. Because the outcomes in this study were continuous variables, MD and 95% CI were used in the forest plots and table descriptions. For continuous variables, the 95% CI was statistically significant if zero was not included. The risk of bias for network meta-analysis was evaluated using confidence in network meta-analysis (CINeMA).

## Results

### Results of studies selection

**Fig. 1:**
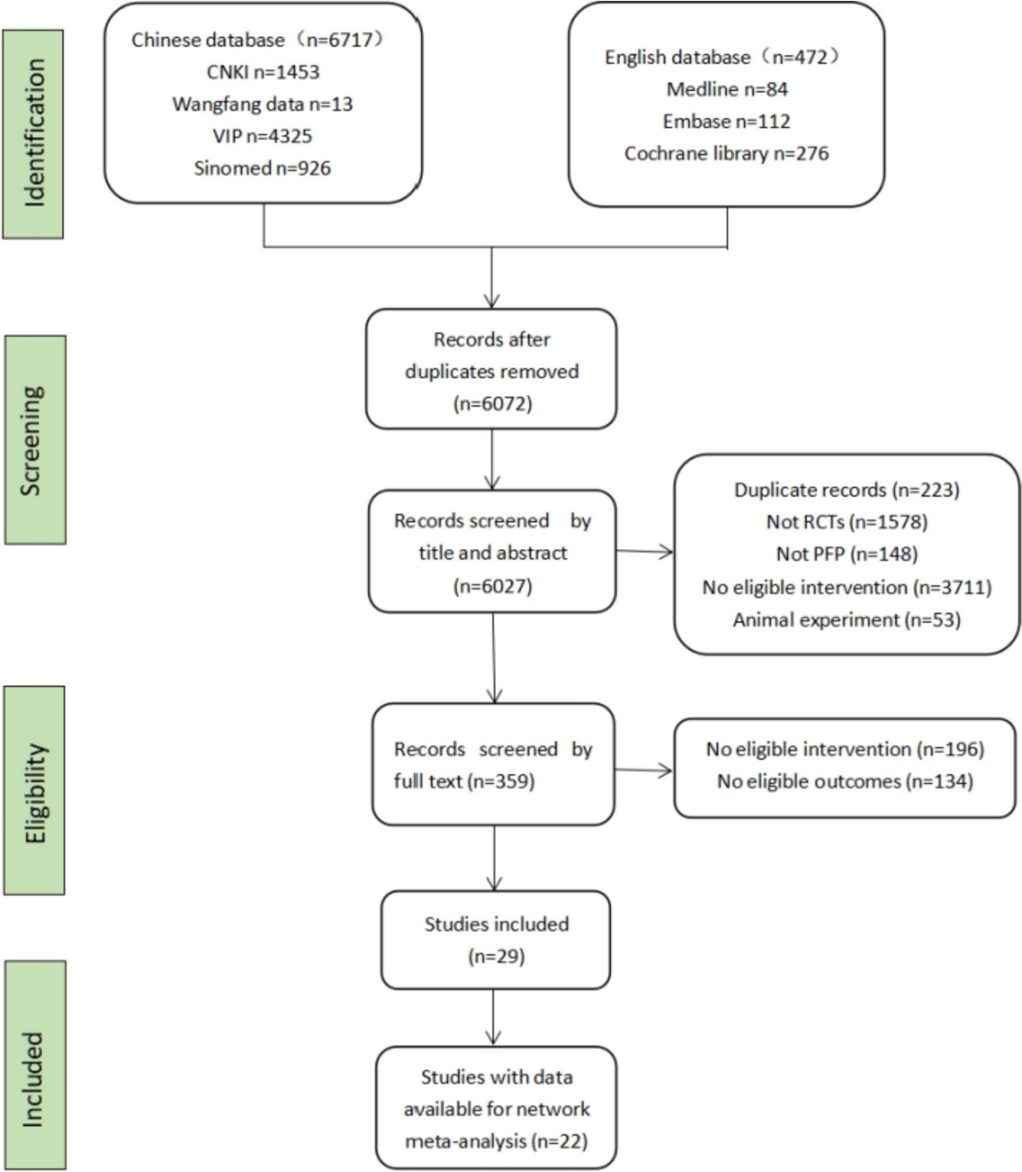
Flowchart of the study process

After a systematic search of four Chinese and three English databases, a total of 7189 studies were identified, among which there were 6717 Chinese studies and 472 English studies. 1117 articles were deleted after using EndnoteX9 software. After reviewing the headlines, abstracts, and full texts, the articles were rigorously screened with respect to the inclusion and exclusion criteria. Studies with inconsistent study types, study subjects, intervention measures, and outcomes were excluded, and 29 articles were retained. Further review of the articles revealed that the outcomes data of seven studies were incorrect, and hence, these were excluded. Finally, 22 trials were retained [26–47], of which 20 were Chinese [26, 27, 29–40, 42–47], and two were English [28, 41]. The flowchart depicting the study selection is shown in Fig. 1.

### Basic characteristics of the included studies

This study included 22 articles with 1814 cases, including 912 males and 742 females (two articles [27, 35] did not report the ratio of males to females). All the articles consisted of two-arm studies with six types of interventions including: acupuncture-only, tuina-only, acupuncture combined with tuina, placebo, single Western medicine, and steroids combined with a Western medicine. MHBN was reported in eight trials [27, 30, 31, 33, 34, 37, 39, 42], SGFS was reported in five trials [28, 29, 41, 46, 47], cure time was reported in six trials [26, 27, 35, 36, 42, 44], Portmann scores was reported in three trials [32, 43, 46], and FDIp was reported in five trials [28, 38, 40, 45, 47]. No adverse events were reported in this study. The characteristics of the included studies have been shown in Table 1, and the demographic characteristics of the studies have been shown in Table 2.

**Table 1:** Basic characteristics of the studies

Study Number	First author, year	duration	Treatment group				Control group				Adverse events reported	Types of outcomes
			Sample	(M/F)	Participant age (years)	Intervention	Sample	(M/F)	Participant age (years)	Intervention		
1	LIU 2012	5w	60	27/33	10–65	C	60	28/32	15–60	A	NR	③
2	QIN2013	30d	50	NR	40.6 ± 19.4	A	50	NR	40.6 ± 19.4	F	NR	① ③
3	Hyo-Jung Kwon 2015	8w	26	12/14	50.85 ± 10.48	A	13	4/9	50.54 ± 11.48	D	NR	② ⑤
4	LV2015	30d	30	14/16	35.4 ± 10.9	B	30	11/19	33.2 ± 11.1	F	NR	②
5	LIAO2015	4w	40	22/18	32.5 ± 11.7	C	40	20/20	32.4 ± 11.7	A	NR	①
6	BAO2015	6w	65	37/28	45.2 ± 17.8	A	65	38/27	44.8 ± 18.2	F	NR	①
7	YU2015	2 months	45	31/14	38.9 ± 6.5	A	45	29/16	38.1 ± 6.47	F	NR	④
8	CHEN2016	20d	45	22/23	47.8 ± 13.6	C	45	25/20	44.3 ± 17.1	A	NR	①
9	XIE2017	10d	68	36/32	39. 22 ± 8. 03	A	68	40/28	39. 54 ± 8. 26	E	NR	①
10	LI2017	10d	30	NR	45.3 ± 1.5	A	30	NR	45.3 ± 1.5	F	NR	③
11	LUO2017	not applicable	40	23/17	44. 5 ± 22. 5	A	40	20/20	45. 5 ± 22. 5	E	NR	③
12	LIN2017	30d	40	20/20	40.23 ± 18.63	A	40	22/18	40.37 ± 18.58	F	NR	①
13	CHEN2017	24d	60	32/28	31.4 ± 1.4	A	60	31/29	31.6 ± 1.9	F	NR	⑤
14	ZOU 2017	2w	30	13/17	36.20 ± 6.80	A	25	11/14	35.40 ± 7.40	F	NR	①
15	MAO 2018	NR	44	31/13	58.90 ± 12.70	A	44	28/16	59.60 ± 11.40	F	NR	⑤
16	Canan Ertemo 2019	4w	20	9/11	40.15 ± 16.19	A	20	9/11	42.70 ± 16.97	D	NR	②
17	HUANG 2020	not applicable	30	17/13	37.69 ± 5.63	A	30	16/14	27.54 ± 5.85	F	NR	① ③
18	XI 2020	1w	55	30/25	36.06 ± 4.91	A	55	31/24	36.44 ± 4.68	E	NR	④
19	XU 2021	not applicable	30	18/12	54.67 ± 3.14	A	30	17/13	53.26 ± 3.52	E	NR	③
20	HUANG 2021	4w	40	29/11	39.61 ± 6.92	A	40	26/14	39.51 ± 7.08	F	NR	⑤
21	ZHENG 2021	4w	40	26/14	41.26 ± 10.23	A	40	24/16	40.87 ± 10.26	E	NR	② ④
22	JIA 2021	2w	29	17/12	50.87 ± 4.73	A	27	16/11	49.35 ± 4.49	F	NR	② ⑤

M: male; F: female; NR: not reported; A: acupuncture-only; B: tuina-only; C: acupuncture + tuina; D: placebo; E: single Western medicine; F: steroids + other Western medicine; ①: MHBN; ②: SFGS; ③: cure time; ④: Portmann scores; and ⑤: FDIp. The same as Table 2

**Table 2:** Demographic characteristics of the studies

Intervention		Proportion
Acupuncture-only		21(47.73%)
Tuina-only		1(2.27%)
Acupuncture + tuina		3(6.81%)
Placebo		2(4.55%)
Single Western medicine		5(11.36%)
Steroids + other Western medicine		12(27.27%)
*Sample size*		
Acupuncture-only	Treatment group	742(80.92%)
	Control group	145(16.16%)
Tuina-only	Treatment group	30(3.27%)
	Control group	0
Acupuncture + tuina	Treatment group	145(15.81%)
	Control group	0
Placebo	Treatment group	0
	Control group	33(3.68%)
Single Western medicine	Treatment group	0
	Control group	233(25.98%)
Steroids + other Western medicine	Treatment group	0
	Control group	486(54.18%)
*Gender*		
Acupuncture-only	Male	454(49.78%)
	Female	358(48.25%)
Tuina-only	Male	14(1.54%)
	Female	16(2.16%)
Acupuncture + tuina	Male	71(7.79%)
	Female	74(9.97%)
Placebo	Male	13(1.43%)
	Female	20(2.70%)
Single Western medicine	Male	132(14.47%)
	Female	101(13.61%)
Steroids + other Western medicine	Male	217(23.79%)
	Female	159(21.43%)
*Duration of treatment*		
Acupuncture-only	≥ 1 month	10(27.78%)
	< 1 month	7(19.44%)
Tuina-only	≥ 1 month	1(2.78%)
	< 1 month	0
Acupuncture + tuina	≥ 1 month	2(5.56%)
	< 1 month	1(2.78%)
Placebo	≥ 1 month	2(5.56%)
	< 1 month	0
Single Western medicine	≥ 1 month	1(2.78%)
	< 1 month	2(5.56%)
Steroids + other Western medicine	≥ 1 month	6(16.67%)
	< 1 month	4(11.11%)
*Time for intervention*		
	Acute phase	6(27.27%)
	According to stages	3(13.64%)
	Without stages	13(59.09%)
*Outcome indicators*		
①		8(29.63%)
②		5(18.52%)
③		6(22.22%)
④		3(11.11%)
⑤		5(18.52%)

### Results of risk of bias

**Fig. 2:**
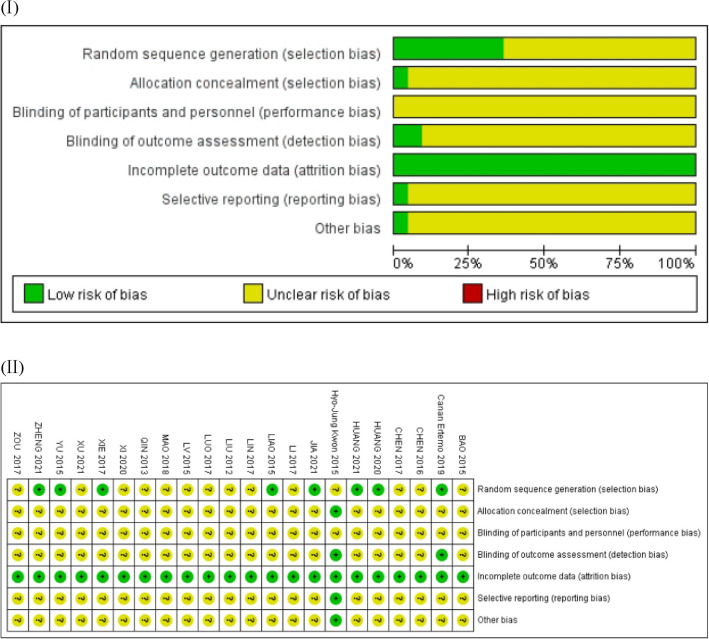
(I) Assessment result of the risk of bias. There are three ratings, green for low risk of bias, yellow for uncertain risk of bias, and red for high risk of bias. Figure 2 (II) is the same. (II) Details of assessment result of risk of bias

Among the 22 studies included, eight studies provided a detailed description of the random sequence generation method [30, 32, 34, 41, 42, 45–47], including the random number table, which provided a low risk of bias, whereas the rest did not specify the randomization method, which resulted in an uncertain risk of bias. Only one study [28] described the allocation concealment, which was rated as a low risk of bias. The blinding of statisticians was mentioned in only two studies [28, 41]. Blinding the clinicians and participants was not possible, considering the specificity of such a trial design. All outcomes were complete, and all selection reports were low risk. Only one trial [28] reported the loss of a case, which was rated as low risk of bias, while the other trials did not include dropouts or lost to follow-up cases. Except for one [28], the other biases in the other studies were uncertain. The assessment result of the risk of bias has been shown in Fig. 2I, and the details of the assessment results have been detailed in Fig. 2II.

### Results of the conventional meta-analysis and the network meta-analysis

#### MHBN

##### Conventional meta-analysis

MHBN was measured in eight studies involving four interventions: A (acupuncture), C (acupuncture + tuina), E (single Western medicine), and F (steroids + other Western medicine). Two studies [30, 33] compared the interventions C and A, five studies [27, 31, 37, 39, 42] compared interventions A and F, and one study [34] compared the interventions A and E.

**Fig. 3:**
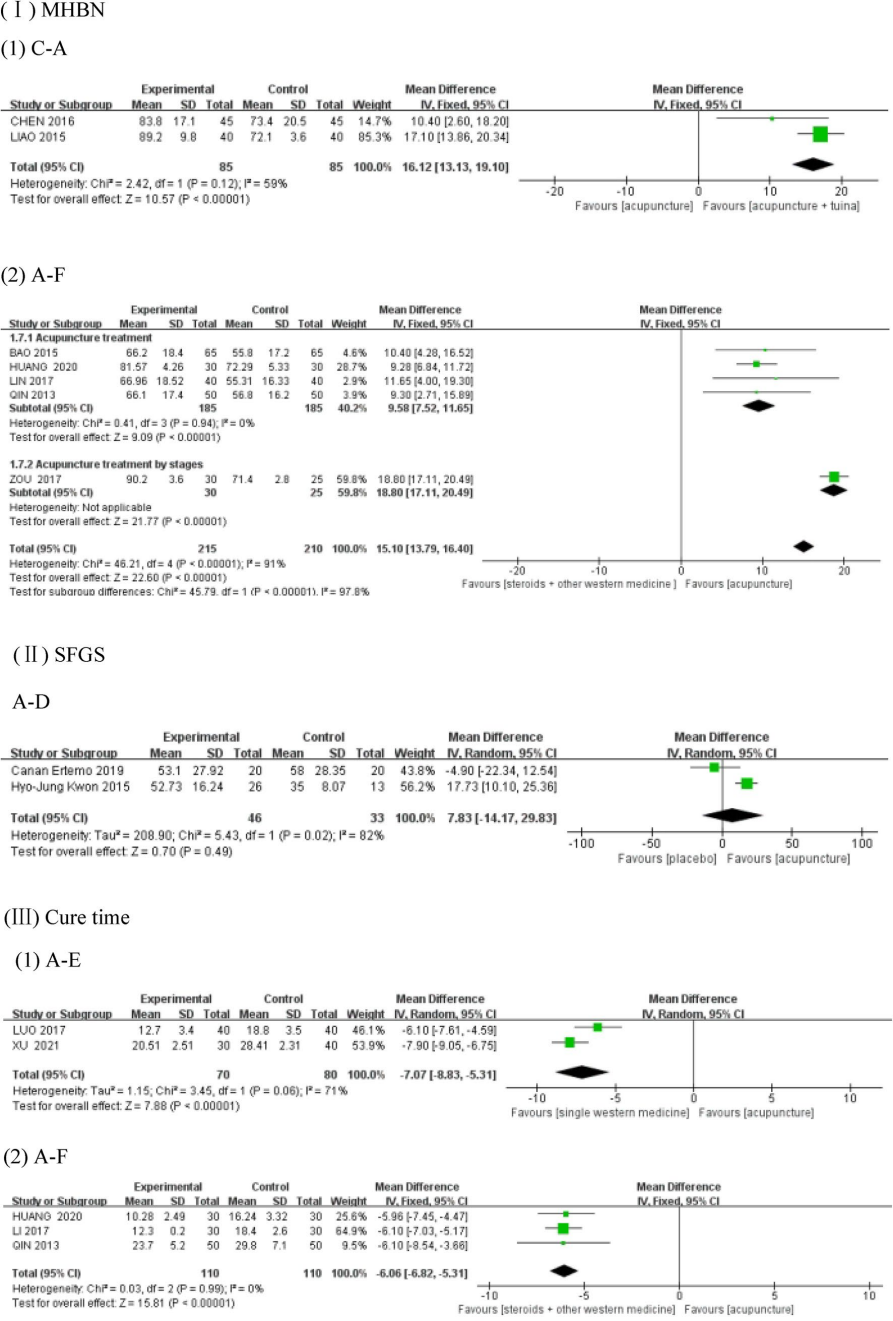

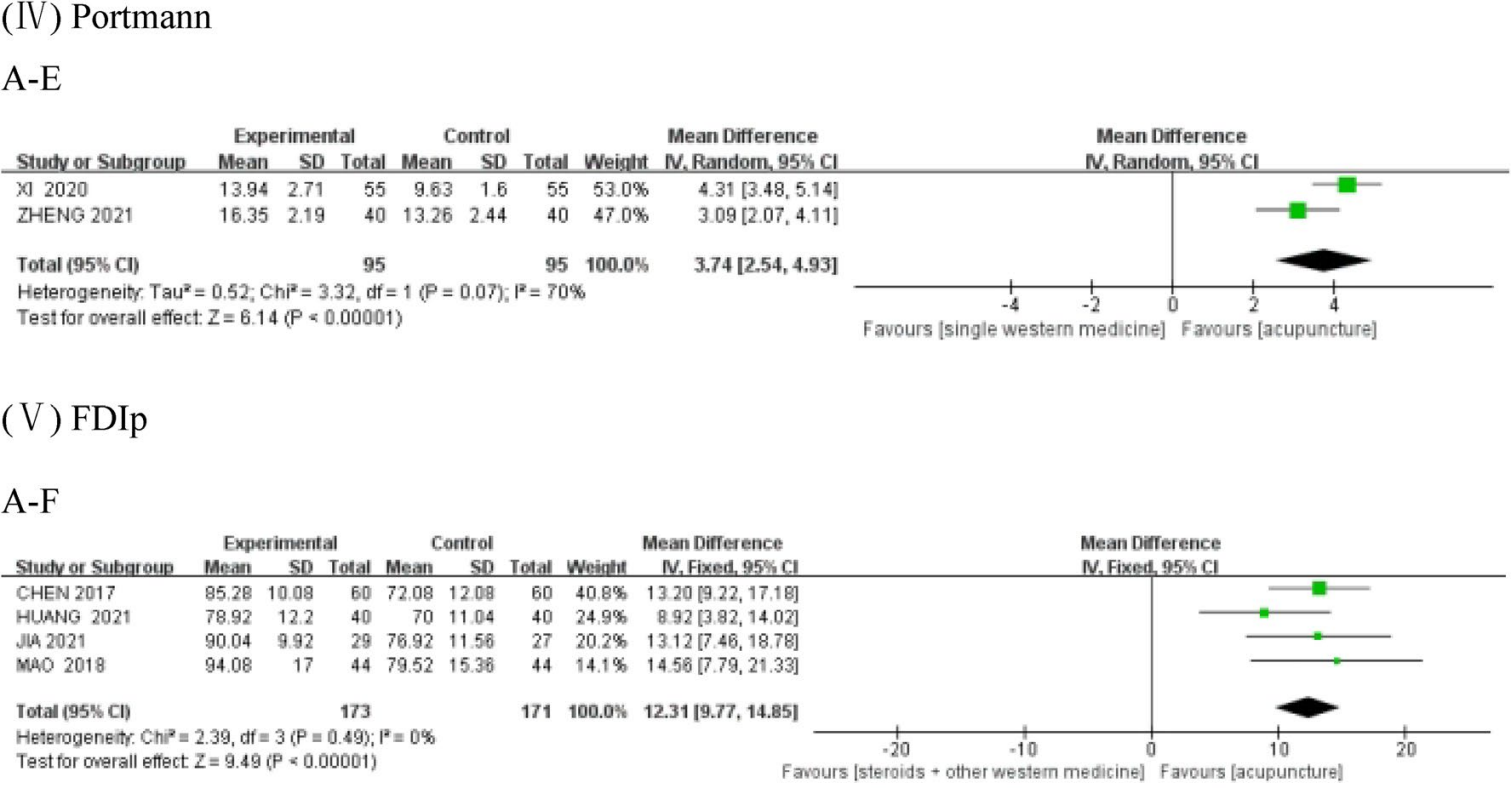
Forest plot of outcomes. A: acupuncture-only; B: tuina-only; C: acupuncture + tuina; D: placebo; E: single Western medicine; and F: steroids + other Western medicine. The same as Fig. 4

Comparisons between interventions C and A using the fixed-effects model (as shown in Fig. 3I–1), resulted in a heterogeneity test score of *P* = 0.12 > 0.1, *I*^2^ = 59%. The forest plot indicated that C was better at improving MHBN than A (MD = 16.12, 95% CI 13.13, 19.10), and the result was statistically significant. With regards to the comparisons between interventions A and F (as shown in Fig. 3I–2), one of the five studies treated patients with PFP phase by phase according to their course of treatment, while the remaining four did not, because of which a subgroup analysis was performed. The total heterogeneity test result was *P* < 0.01, *I*^2^ = 91%. The heterogeneity test result of the subgroup without staged acupuncture treatment using the fixed-effects model was *P* = 0.94 > 0.1, *I*^2^ = 0. The forest plot demonstrated the superiority of A over F (MD = 15.10, 95% CI 13.79, 16.40) in improving MHBN, and the result was statistically significant. Further details can be viewed in Table 3.

**Table 3:** Results of the conventional meta-analysis

Outcomes	Comparison of intervention	Number of studies	Number of patients	MD	95% CI
MHBN	C-A	2	170	16.21	13.13, 19.10*
	A-F	5	425	15.10	13.79, 16.40*
SFGS	A-D	2	79	7.83	− 14.17, 29.83
Cure time	A-E	2	150	− 7.07	− 8.83, − 5.31*
	A-F	3	220	− 6.06	− 6.82, − 5.31*
Portmann scores	A-E	2	190	3.74	2.54, 4.93*
FDIp	A-F	4	344	12.31	9.77, 14.85*

*The difference was statistically significant. A: acupuncture-only; B: tuina-only; C: acupuncture + tuina; D: placebo; E: single Western medicine; F: steroids + other Western medicine. The same as Tables 4, 5, 6, 7, 8

##### Network meta-analysis

**Fig. 4:**
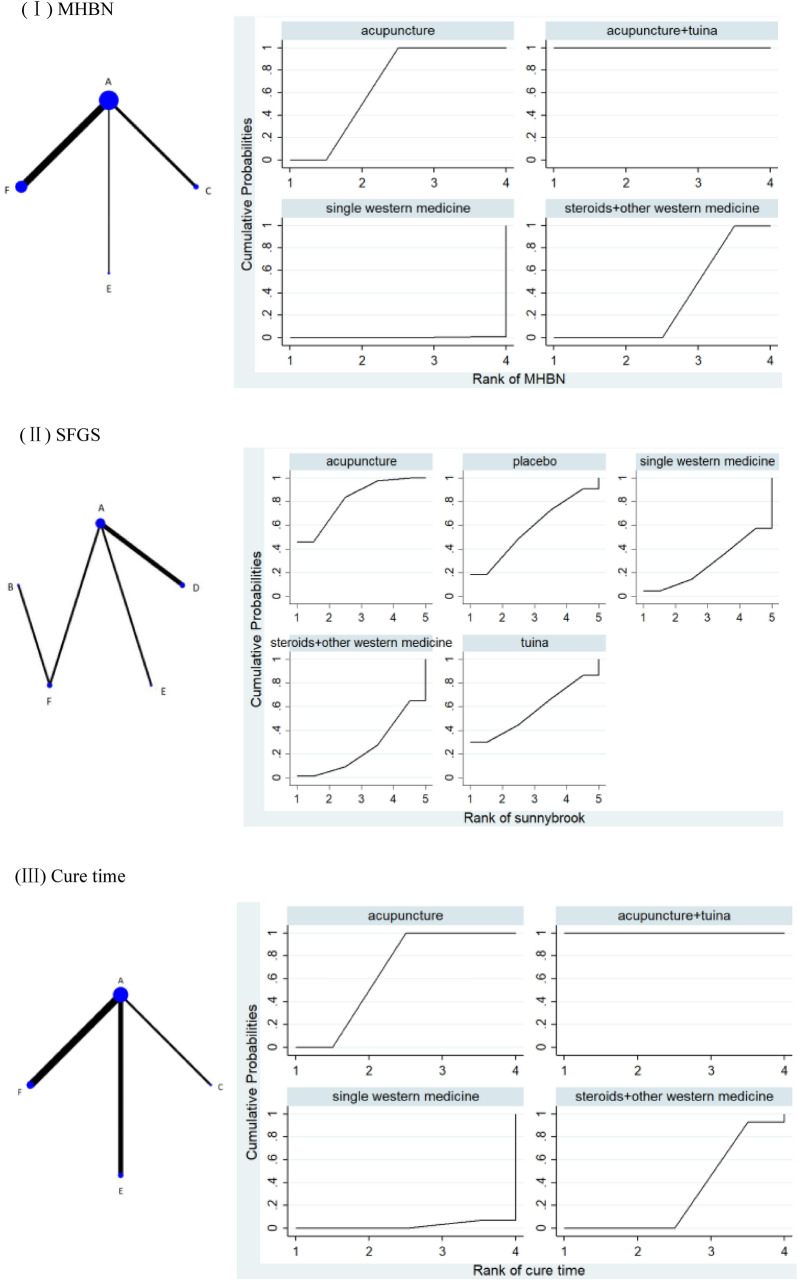

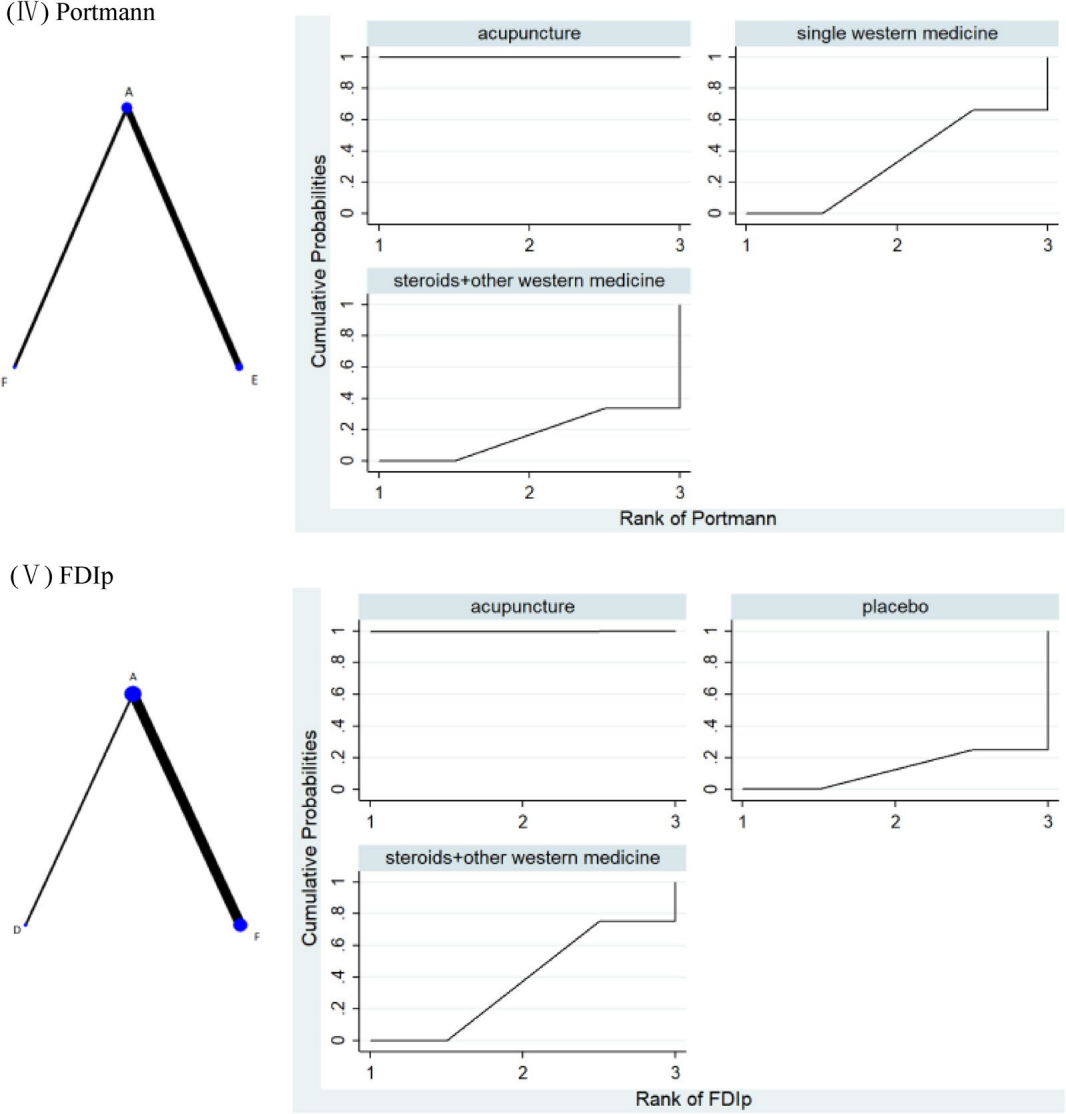
Network plot (left) and SUCRA (right) of outcomes. Node represents the intervention, and node size is positively correlated with the total sample size of the intervention. The line represents a direct comparison between the two interventions linked, and the line thickness is positively correlated with the number of studies on the two interventions

The network plot indicated the absence of the formation of closed loops, as shown in Fig. 4I. Results of the consistency analysis indicated that PSRF was equal to or close to 1, demonstrating good data convergence. A total of six pairwise comparison items were observed in the network meta-analysis. The probability ranking of SUCRA for the four interventions demonstrated that C ranked the highest at 100%, A ranked second at 66.7%, F ranked third at 33.1%, and E ranked fourth at 0.2%. Observation of the data in Table 4 indicated that C was better than A (MD = 14.53, 95%CI 7.57,21.49), F (MD = 26.85, 95%CI 18.62,35.08), and E (MD = 38.80, 95%CI 27.92,49.69) in improving MHBN; A was better than F (MD = 12.32, 95%CI 8.02,16.61) and E (MD = 24.27, 95%CI 15.90,32.65) in improving MHBN; and F was better than E (MD = 11.96, 95%CI 2.55,21.36) in improving MHBN. The differences were statistically significant. Higher MHBN corresponds to better facial neural function. Therefore, the comparative probability of intervention measures in improving the MHBN score was ranked as C > A > F > E. The rank probability is shown in Fig. 4I.

**Table 4:** Results of the network meta-analysis of MHBN

Acupuncture + tuina	Acupuncture-only	Steroids + other Western medicine	Single Western medicine
0			
14.53 (7.57,21.49)*	0		
26.85 (18.62,35.08)*	12.32 (8.02,16.61)*	0	
38.80 (27.92,49.69)*	24.27 (15.90,32.65)*	11.96 (2.55,21.36)*	0

Statistically significant differences are highlighted in bold

#### SFGS

##### Conventional meta-analysis

Five studies involving five interventions: A (acupuncture), B (tuina), D (placebo), E (single Western medicine), and F (steroids + other Western medicine) reported in terms of SFGS. Two studies [28, 41] compared A and D, one study [29] compared B and F, one study [46] compared A and E, and one study [47] compared A and F.

In the comparison between A and D, using the random-effects model (as shown in Fig. 3II), the heterogeneity test was *P* = 0.02, *I*^2^ = 82% > 50. The forest plot indicated the lack of statistical significance between A and D (MD = 7.83, 95%CI  − 14.17, 29.83) in improving SFGS. Details of the same have been given in Table 3.

##### Network meta-analysis

The network plot indicated no formation of closed loops, as evident from Fig. 4II. Results of the consistency analysis demonstrated good data convergence, with PSRF being equal to or close to 1. A total of ten pairwise comparison items were generated in the network meta-analysis. The probability ranking of SUCRA for the five interventions demonstrated that A ranked the highest at 81.8%, D ranked second at 57.7%, B ranked third at 57.0%, E ranked fourth at 27.9%, and F ranked fifth at 25.7%. Observation of the data in Table 5 indicated the absence of statistical significance between A and D (MD = 7.83, 95%CI  − 14.49, 30.16), B (MD = 9.14, 95%CI − 31.88, 50.16), E (MD = 21.06, 95%CI -7.46, 49.58), and F (MD = 20.75, 95%CI − 7.87, 49.36) in improving SFGS. No statistical significance was observed between D and B (MD = 1.31, 95%CI − 45.39, 48.00), E (MD = 13.23, 95%CI -22.99, 49.45), F (MD = 12.92, 95%CI − 23.38, 49.21). There was no statistical significance between B and E (MD = 11.92, 95%CI − 38.04, 61.88) and F (MD = 11.61, 95%CI − 17.79, 41.00). No statistical significance was observed between E and F (MD = − 0.31, 95%CI − 40.71, 40.09) in improving SFGS. A higher SFGS corresponds to better facial nerve function. Therefore, the comparative probability ranking of intervention measures to improve SFGS was A > D > B > E > F. The rank probability is shown in Fig. 4II.

**Table 5:** Results of the network meta-analysis of SFGS

Acupuncture-only	Placebo		Tuina-only		Single Western medicine		Steroids + other Western medicine	
0								
7.83 ( − 14.49,30.16)	0							
9.14 ( − 31.88,50.16)	1.31 ( − 45.39,48.00)		0					
21.06 ( − 7.46,49.58)	13.23 ( − 22.99,49.45)		11.92 ( − 38.04,61.88)		0			
20.75 ( − 7.87,49.36)	12.92 ( − 23.38,49.21)		11.61 ( − 17.79,41.00)		− 0.31 ( − 40.71,40.09)		0	

#### Cure time

##### Conventional meta-analysis

Six studies involve four interventions: A (acupuncture), C (acupuncture + tuina), E (single Western medicine), and F (steroids + other Western medicine) reported the cure time. Three studies [25, 33, 40] compared A and F, two studies [36, 44] compared interventions A and E, and one study [26] compared interventions C and A.

Comparisons between interventions A and E using the random-effects model (as shown in Fig. 3 (III)-(1)), resulted in the heterogeneity test score of *P* = 0.06, *I*^2^ = 71% > 50%. The forest plot demonstrated the superiority of A over E (MD = -7.07, 95%CI − 8.83, − 5.31) in shortening the cure time, with the difference being statistically significant. When interventions A and F were compared using the fixed-effects model (as shown in Fig. 3III-2), the heterogeneity test was *P* = 0.99 > 0.1, *I*^2^ = 0%. The forest plot indicated that A was superior to F (MD = − 6.06, 95%CI − 6.82, − 5.31) in shortening the cure time, and the difference was statistically significant. Details can be viewed in Table 3.

##### Network meta-analysis

The network plot showed that there was no closed loop formation, as evident from Fig. 4III. Consistency analysis results indicated good data convergence with PSRF being equal to or close to 1. The network meta-analysis generated a total of six pairwise comparison items. The probability ranking of SUCRA for the four interventions indicated that C ranked highest at 100%, A ranked second at 66.7%, F ranked third at 31.0%, and E ranked fourth at 2.3%. The data given in Table 6 indicated that C was superior to A (MD = -6.09, 95%CI − 7.70,-4.49), F (MD = − 12.15, 95%CI − 13.98, + 10.33), and E (MD = − 13.25, 95%CI − 15.19, − 11.30) in shortening the cure time; A was better than F (MD = − 6.06, 95%CI − 6.94, − 5.18) and E (MD = − 7.15, 95%CI − 8.25,-6.05) in shortening the cure time, with the differences being statistically significant. No statistical significance was observed between F and E (MD = − 1.09, 95%CI − 2.50, 0.31). Shorter cure times indicated better outcomes. Therefore, the comparative probability ranking of intervention measures to shorten the cure time was C > A > F > E. The rank probability is shown in Fig. 4III.

**Table 6:** Results of the network meta-analysis of the cure time

Acupuncture + tuina	Acupuncture-only		Steroids + other Western medicine		Single Western medicine	
0						
− 6.09 ( − 7.70, − 4.49)*	0					
− 12.15 ( − 13.98, − 10.33)*	− 6.06 ( − 6.94, − 5.18)*		0			
− 13.25 ( − 15.19, − 11.30)*	− 7.15 ( − 8.25, − 6.05)*		− 1.09 ( − 2.50,0.31)		0	

Statistically significant differences are highlighted in bold

#### Portmann scores

##### Conventional meta-analysis

Portmann scores was reported in three studies involving three interventions: A (acupuncture), E (single Western medicine), and F (steroids + other Western medicine). Two studies [43, 46] compared A and E, and one study [32] compared A and F.

In the comparison between A and E using the random-effects model (as shown in Fig. 3IV), the heterogeneity test was *P* = 0.07, *I*^2^ = 70% > 50%. The forest plot demonstrated that A was superior to E (MD = 3.74, 95%CI 2.54, 4.93) in improving Portmann scores, and the difference was statistically significant. Details are shown in Table 3.

##### Network meta-analysis

The network plot indicated the absence of the closed loop form, as shown in Fig. 4IV. The results of the consistency analysis demonstrated good data convergence with PSRF values equal to or close to 1. A total of three pairwise comparison items were generated in the network meta-analysis. Probability ranking of SUCRA for the three interventions demonstrated that A ranked first at 99.9%, E ranked second at 33.2%, and F ranked third at 16.9%. Data in Table 7 have demonstrated that A was superior to E (MD = 3.74, 95%CI 2.54, 4.93), F (MD = 4.34, 95%CI 1.52, 7.16) in improving Portmann scores, and the difference was statistically significant. There was no statistical significance between E and F (MD = 0.60, 95%CI − 2.46,3.67). Higher Portmann scores indicated better facial nerve function. Therefore, the comparative probability ranking of intervention measures to improve Portmann scores was A > E > F. The rank probability is shown in Fig. 4IV.

**Table 7:** Results of the network meta-analysis of Portmann scores

Acupuncture-only	Single Western medicine	Steroids + other Western medicine
0		
3.74 (2.54,4.93)*	0	
4.34 (1.52,7.16)*	0.60 ( − 2.46,3.67)	0

Statistically significant differences are highlighted in bold

#### FDIp

##### Conventional meta-analysis

Five studies involve three interventions: A (acupuncture), D (placebo), and F (steroids + other Western medicine) reported FDIp Index. Four studies [38, 40, 45, 47] compared A and F, and one study [28] compared A and D.

In the comparison between A and F using the fixed-effects model (as shown in Fig. 3V), the heterogeneity test was *P* = 0.49 > 0.1, *I*^2^ = 0%. The forest plot indicated the superiority of A over F (MD = 12.31, 95%CI 9.77, 14.85) in improving FDIp, and the difference was statistically significant. Details can be seen in Table 3.

##### Network meta-analysis

The network plot showed that there was no closed loop form, as shown in Fig. 4V. Results of the consistency analysis indicated good data convergence with PSRF being equal to or close to 1. A total of three pairwise comparison items were generated in the network meta-analysis. The probability ranking of SUCRA for the three interventions showed that A ranked first at 99.8%, F ranked second at 37.5%, and D ranked third at 12.7%. The data in Table 8 indicated that A was superior to F (MD = 12.31, 95%CI 9.77, 14.85) and D (MD = 16.74, 95%CI 4.35, 29.13) in improving FDIp, and the difference was statistically significant. There was no statistical significance between F and D (MD = 4.43, 95%CI − 8.22, 17.08). Higher FDIp Index corresponded to better outcomes. Therefore, the comparative probability ranking of intervention measures to improve FDIp was A > F > D. The rank probability has been shown in Fig. 4V.

**Table 8:** Results of the network meta-analysis of FDIp

Acupuncture-only	Steroids + other Western medicine	Placebo
0		
12.31 (9.77,14.85)*	0	
16.74 (4.35,29.13)*	4.43 (-8.22,17.08)	0

Statistically significant differences are highlighted in bold

### Results of sensitivity analysis

**Fig. 5:**
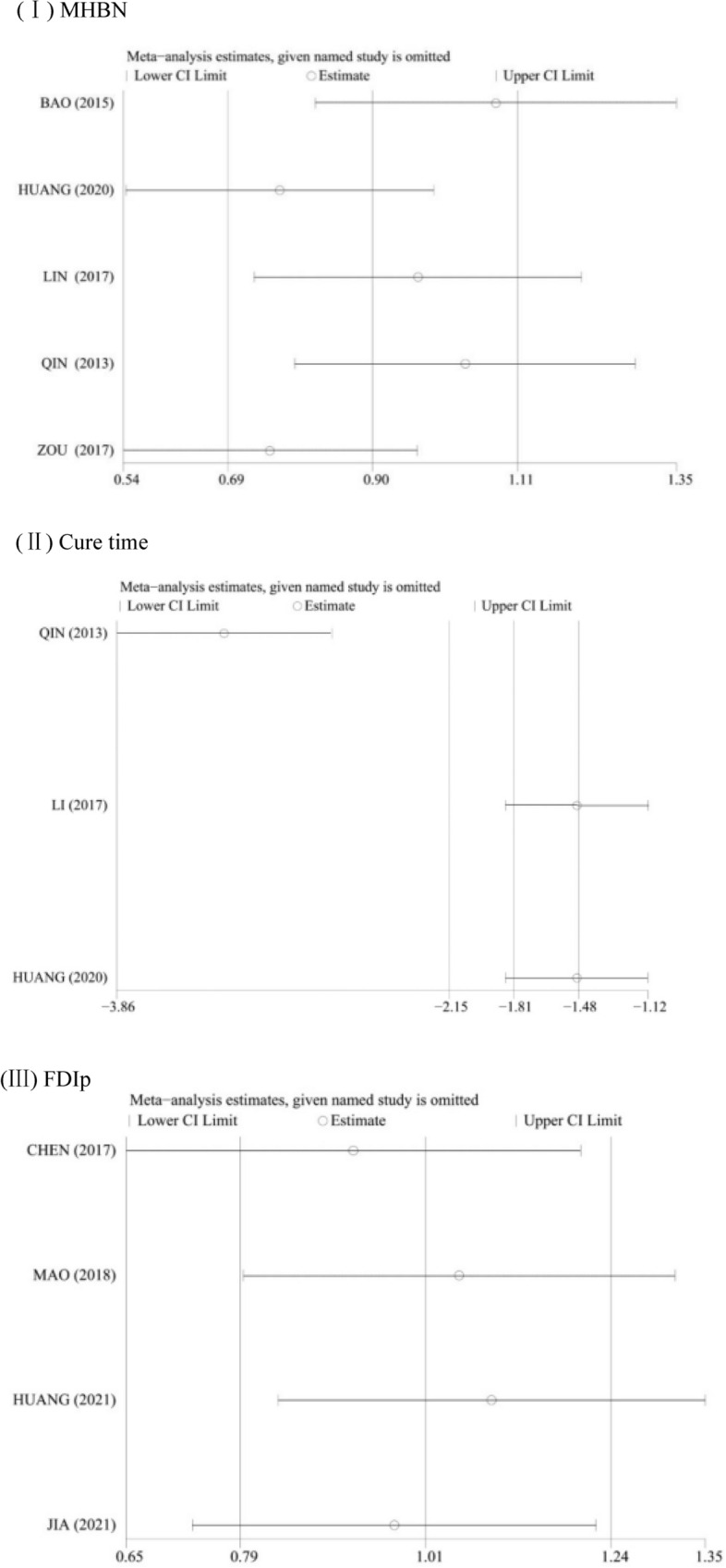
Results of sensitivity analysis

Sensitivity analysis was aimed at exploring the impact of the excluded studies on the combined effect value of other studies by eliminating studies one by one. Due to the limited number of articles among interventions, sensitivity analysis was only performed on MHBN, cure time, and FDIp to explore the stability of the meta-analysis. As evident from Fig. 5, the meta-analysis results of MHBN and FDIp were more stable, whereas the result of cure time was less stable.

### Results of Risk of bias of network meta-analysis

**Fig. 6:**
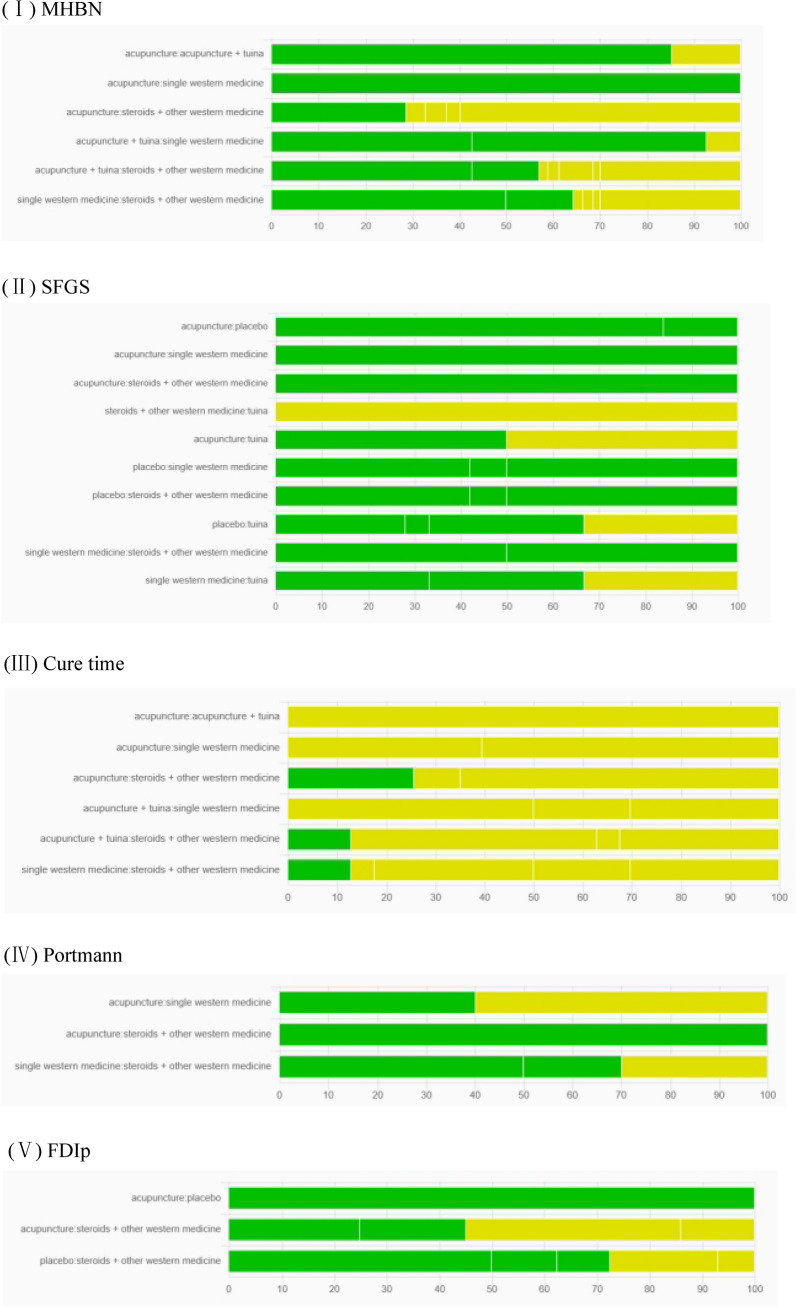
Risk of a bias bar chart for network meta-analysis. Each bar represents a relative treatment effect estimated from the network meta-analysis. Each bar shows the percentage contribution from studies judged to be at low (green), moderate (yellow), and high (red) risk of bias

CINeMA [48] was used for assessing the risk of bias in the studies included in the network meta-analysis by combining the contributions of the studies and the risk of bias judgments. As shown in Fig. 6, each bar represented the percentage contribution from the studies and was judged to be at low (green), moderate (yellow), and high (red) risk of bias. The percentage contributions from the comparison between A and F (71.27%) in MHBN, B and F (100%) in SFGS, A and E (59.87%) in Portmann scores, and A and F (54.94%) in FDIp at moderate risk of bias were large, indicating study limitations. Comparisons between A and C (100%), A and E (100%), A and F (74.38%), C and E (100%), C and F (87.19%), and E and F (87.19%) in cure time at moderate risk of bias indicated some concern about limitations.

## Discussion

### Summary

This study included 22 articles involving 1814 patients diagnosed with PFP, without any restrictions on the duration and phase of the disease. The five outcomes included in this study reflect the level of dynamic and static facial neural functions and the course of treatment. The primary outcomes were MHBN, and SFGS and the secondary outcomes were cure time, Portmann scores, and FDIp, using conventional meta-analysis and network meta-analysis.

The results of the conventional meta-analysis were as follows (Table 3): ① Acupuncture + tuina was better than acupuncture-only (MD = 16.12, 95% CI 13.13, 19.10), and acupuncture-only was better than steroids + other Western medicine (MD = 15.10, 95% CI 13.79, 16.40) concerning improvement in MHBN. ② In terms of improving SFGS, no statistical significance was observed between acupuncture-only and placebo (MD = 7.83, 95% CI − 14.17, 29.83). ③ Acupuncture-only was better than single Western medicine (MD = − 7.07, 95% CI − 8.83, − 5.31) and steroids + other Western medicines (MD = -6.06, 95% CI − 6.82, − 5.31) in terms of reducing the course of the treatment. ④ Acupuncture-only was better than single Western medicine (MD = 3.74, 95% CI 2.54, 4.93) in terms of improving Portmann scores. ⑤ Acupuncture-only was better than steroids + other Western medicine (MD = 12.31, 95% CI 9.77, 14.85) in terms of improving FDIp. The application of acupuncture in the treatment of PFP was observed to effectively promote the recovery of facial nerve function, shorten the course of treatment, and have a better effect compared with single Western medicine and steroids + other Western medicine.

The results of the network meta-analysis were as follows: ① Acupuncture + tuina was most likely the optimal therapy in terms of improving MHBN (SUCRA was 100%); acupuncture + tuina was more effective than acupuncture-only (MD = 14.53, 95% CI 7.57,21.49), steroids + other Western medicine (MD = 26.85, 95% CI 18.62,35.08), and single Western medicine (MD = 38.80, 95% CI 27.92,49.69) for treating PFP. ② With respect to improving SFGS, acupuncture was most likely the optimal therapy (SUCRA was 81.8%). However, no statistically significant difference was observed between acupuncture and placebo (MD = 7.83, 95% CI -14.49, 30.16), tuina (MD = 9.14, 95% CI − 31.88, 50.16), single Western medicine (MD = 21.06, 95%CI − 7.46, 49.58), and steroids + other Western medicine (MD = 20.75, 95% CI − 7.87, 49.36). ③ In terms of reducing the course of treatment, acupuncture + tuina was the most likely to be optimal therapy (SUCRA was 100%), when compared with acupuncture-only (MD = -6.09, 95% CI − 7.70, − 4.49), steroids + other Western medicine (MD = − 12.15, 95%CI − 13.98,-10.33), and single Western medicine (MD = − 13.25, 95% CI − 15.19, − 11.30). ④ The most likely optimal therapy in terms of improving Portmann scores was acupuncture-only (SUCRA was 99.9%), better than single Western medicine (MD = 3.74, 95% CI 2.54, 4.93) and steroids + other Western medicine (MD = 4.34, 95% CI 1.52, 7.16). ⑤ Acupuncture-only was the most likely optimal therapy for improving FDIp (SUCRA was 99.8%), better than steroids + other Western medicine (MD = 12.31, 95% CI 9.77, 14.85) and placebo (MD = 16.74, 95% CI 4.35, 29.13). Overall, it was observed that acupuncture + tuina and acupuncture-only are more effective for treating PFP compared with Western medicine (taken alone or in combination). TCM therapies are definitely superior to Western medicine and can effectively improve the clinical symptoms of PFP. This result is consistent with the results obtained in several other studies conducted in China as well as abroad [49, 50], but the currently available evidence is not sufficient to decide the optimal treatment modality for treating PFP.

### Explore heterogeneity

① Meta-analysis of MHBN indicated a high heterogeneity test of A and F (*P* < 0.01, *I*^2^ = 91%). A careful research of these five studies revealed that in one of the studies, acupuncture was applied based on the stage of PFP; that is, acupuncture was applied differently during the acute stage, the resting stage, and the recovery stage. The remaining four studies had lower heterogeneity (*P* = 0.94, *I*^2^ = 0%). The administration of acupuncture treatment in accordance with the stage of PFP in one study could have been the reason for the high heterogeneity. ② Meta-analysis of SFGS revealed a high heterogeneity between A and D (*P* = 0.02, *I*^2^ = 82%). It was found that one study included patients who had a course of illness for at least six months and were treated for eight weeks, and the other study included patients who had a course of illness for three months and were treated for four weeks. The variation in the duration of illness and treatment in the two studies could possibly have contributed to the high heterogeneity. ③ Meta-analysis of cure time indicated high heterogeneity was high in the comparison between A and E (*P* = 0.06, *I*^2^ = 71%). Careful scrutiny indicated that one of the studies treated patients with PFP in the acute stage with acupuncture, and the duration of the disease was not specified in the other study, which could possibly explain the high heterogeneity. ④ Meta-analysis of Portmann scores demonstrated elevated results of the heterogeneity test when comparing A and E (*P* = 0.07, *I*^2^ = 70%). Further research indicated that one study treated patients with PFP in the acute phase and the other did not constrain the stage of the disease course, which could have contributed to the high heterogeneity.

### Interpretation

Western medical treatment options are limited for PFP of self-limited disease and are often helpless in the face of severe sequelae and refractory facial paralysis. The commonly used drugs for treating PFP include prednisone, acyclovir, and mecobalamin. Some scholars believe that PFP that remains unhealed for more than three months is called refractory facial paralysis [51]; hence, early intervention and treatment are recommended to shorten the course of the disease [52].

TCM therapy has a long history, and in combination with innovation and modern medicine, it has developed into an important complementary therapy that is believed to be safe, effective, and diverse. Acupuncture in combination with other therapies is the dominant TCM treatment modality in the treatment of PFP, which is applied to all phases of facial paralysis and also has been found to be significantly effective in treating refractory facial paralysis [53, 54]. Some previously conducted studies have demonstrated that acupuncture can increase blood perfusion volume and promote microcirculation [55]. TCM believes that the pathological changes of PFP are concentrated in the facial muscles and that acupuncture, tuina, and other external treatments can eliminate the condition and stimulate the body’s health qi for speedy recovery of the facial nerves. For exploring the clinical effect of acupuncture combined with tuina, a RCT conducted by Fang et al. [56] compared the efficacies of groups treated with acupuncture combined with tuina, acupuncture-only, and tuina-only, and the results indicated that the effective rate of acupuncture combined with tuina group was 97.8%, which was significantly higher than that of acupuncture-only group (86.9%) and tuina-only group (84.8%). In addition to combined therapy with tuina and moxibustion [57], electric stimulation [58], cupping [59], and fire needles [60], all possess unique therapeutic advantages. In terms of clinical applications, combination therapies are preferred over single therapies and hold more promise. A meta-analysis conducted by Ye et al. [61] explored the efficacy of acupuncture combined with comprehensive therapy in the treatment of PFP, and the results indicated that acupuncture combined with comprehensive therapy was superior to acupuncture-only. A study conducted by Liu [62] involved the application of different acupuncture treatments based on the phase of PFP in the acute phase, stable phase, and convalescent phase. Following three courses of acupuncture, the symptoms of the patients in the treated group showed gradual improvement, and facial nerve function scores increased significantly compared to the control group who were prescribed oral Western medicines.

Our present study demonstrated that the included interventions (acupuncture-only and acupuncture combined with tuina) were effective in treating PFP. The 95% CI of MD in the results of the network meta-analysis of each intervention was wide, which could have been related to the limited number of articles and smaller sample sizes of the comparative interventions included in this study, which could have led to reduced statistical power. The ranking of each intervention in terms of improved facial neural function scores varied widely, because of which, an optimal treatment approach could not be specified. There were fewer studies involving acupuncture combined with tuina and tuina-only than acupuncture-only therapy in the studies included in this meta-analysis. In addition, clinical efficacy assessment systems for PFP have not yet been developed and the lack of consensual norms to be followed when performing RCT could lead to certain errors.

### Strengths and limitations

The advantage of this study lies in the detailed analysis based on conventional meta-analysis and network meta-analysis of acupuncture combined with tuina and acupuncture-only in the treatment of PFP. In addition, the cumulative probable ranking of the best intervention measures in terms of improving each outcome was given.

This study has some limitations: (1) Only English and Chinese databases were searched, and the number of trials included was limited. All studies included two-arm trials with a modest number of participants. (2) The overall quality of the articles included in the study was not optimal, and the quality of distribution hiding and blind implementation in most articles was low, and the risk of bias was large. (3) None of the included trials reported adverse events, and this study failed to analyze the safety of each intervention. (4) The lack of large sample RCTs in this study could have increased the sampling error, resulting in a possibly compromised statistical efficacy.

## Conclusion

The results of the comprehensive analysis presented in this study demonstrate that first, acupuncture in combination with tuina can improve the clinical symptoms, promote the recovery of facial nerve, and shorten the treatment time. In addition to acupuncture, tuina has a tendency to increase the curative effect. Second, acupuncture combined with tuina, acupuncture-only, and Western medicine (used alone or in combination) can effectively improve facial nerve function. However, the current evidence does not demonstrate that acupuncture combined with tuina is more effective than acupuncture-only, nor does it recommend it as the best treatment plan. It is recommended to conduct higher-quality clinical studies, form a therapeutic effect assessment system, unify clinical treatment guidelines, and explore the optimal treatment of PFP.
